# Does the type of electrode affect the electromyoneurographic parameters in rats?^1^

**DOI:** 10.1590/s0102-865020190030000004

**Published:** 2019-03-18

**Authors:** Danusa Neves Somensi, Renan Kleber Costa Teixeira, Daniel Haber Feijó, Karine Drumond Loureiro, André Lopes Valente, Luan Teles Ferreira de Carvalho, Faustino Chaves Calvo, Deivid Ramos dos Santos, Rui Sergio Monteiro de Barros

**Affiliations:** I MD, MS, Department of Neurology, School of Medicine, Universidade do Estado do Pará (UEPA), Belem-PA, Brazil. Conception, design, intellectual and scientific content of the study; interpretation of data; manuscript writing; critical revision.; II MD, MS, Department of Experimental Surgery, School of Medicine, UEPA, Belem-PA, Brazil. Interpretation of data, manuscript writing, critical revision.; III Fellow Master degree, Postgraduate Program in Surgery and Experimental Research, School of Medicine, UEPA, Belem-PA, Brazil. Acquisition and interpretation of data, manuscript writing.; IV MD, Department of Neurology, School of Medicine, UEPA, Belem-PA, Brazil. Acquisition and interpretation of data.; V Graduate student, School of Medicine, UEPA, Belem-PA, Brazil. Acquisition and interpretation of data.; VI PhD, Associate Professor, Department of Experimental Surgery, School of Medicine, UEPA, Belem-PA, Brazil. Scientific content of the study, critical revision.

**Keywords:** Neurophysiology, Neural Conduction, Experimental Development, Rats

## Abstract

**Purpose:**

To evaluate if the type of electrode (needle vs. surface) affects the electromyoneurography parameters in rats.

**Methods:**

Twenty male rats were anesthetized, then compound muscle action potential were recorded using a Neuropack S1 MEB- 9400^©^. All animals were submitted to two electroneuromyography analysis: first with surface electrode and then by needle electrode. We evaluated the latency, amplitude, duration and area of the negative peak of the gastrocnemius and cranial tibial muscles.

**Results:**

There were no significant differences between the groups in the mean of duration, latency, amplitude or area of the negative peak in gastrocnemius and cranial tibial muscles.

**Conclusion:**

The type of electrode does not affect the electroneuromyography parameters.

## Introduction

 Functional recovery is the ultimate goal of treatments that aim peripheral nerve recovery, constituting a major challenge in modern rehabilitation medicine^1^. The evaluations of the nervous parameters are very complex, and these nuances must be present in the experimental models, which aim to simulate pathologies of the peripheral nervous system, needing to be consistent, obtaining reliable parameters to the clinical correlation^1^ ^-^ ^4^.

In the case of traumatic lesions of the peripheral nerves with functional loss, the electrophysiological evaluation has a fundamental participation in the analysis of the obtained results^2^. Several factors such as age, sex, muscle, electrode type, sensitivity, stimulus frequency, frequency filters, temperature change and humidity may interfere with the responses to electrostimulation. However, the literature lacks studies that analyze what factors and how they influence the data obtained^1^ ^,^ ^3^ ^,^ ^4^.

The various experimental studies that evaluate this parameter use adaptations of the existing human model^3^, performed according to the experimental animal, the nerve to be studied and the type of the experiment, seeking the maximum homogeneousness of the factors to be studied. Frequently, due to its invasive characteristic, requires euthanasia of the animal and makes it difficult to follow the animals in the study^1^.

The standardization of parameters refers to the stimulator, preamplifier and oscilloscope, maintenance of the ambient temperature, maintenance of the body temperature of the animal, and application of mineral oil to avoid the drying of the exposed structures^4^ ^,^ ^5^. However, the literature lacks studies that aim to verify the effects of a change in some of these parameters^4^ ^-^ ^7^, such as the type of electrode used, which may be surface or needle. Therefore, we aim to assess if the type of electrode (needle Vs surface) affects the electromyoneurography parameters in rats.

## Methods

 The study followed the rules set out in the Brazilian Law for Animal Care (Law: 11.794/08) and the project was approved in advance by the Animal Use and Care Committee at the Universidade do Estado do Pará (protocol 19/2015). Twenty male Wistar rats (*Rattus norvegicus*) were obtained from the Animal House at the Experimental Surgery Laboratory, and kept in a controlled environment with food and water *ad libitum*. 

All animals were submitted to the same compound muscle action potential recording. The analysis was performed in the same day on the morning, in a room with controlled environment. The temperature was set on 24ºC. The electrophysiologic testing protocol was the following: 1) The rats were anesthetized with intraperitoneal ketamine (70 mg/kg) and xylazine (10 mg/kg)^8^ ^,^ ^9^ and then shaved and placed in a horizontal supine position. 2) The temperature was measured with infrared thermometer at the time of examination. 3) The standard electrodes were placed at the same position: ground electrode in right lateral back of animal; reference electrode in the second interdigit of right paw; and active electrode in the bulk of tibial cranial/ and gastrocnemius muscle. The stimulation was performed on the right sciatic notch with intensity of 10mA and duration of 0.2ms. 4) All animals were submitted to two electroneuromyography analysis: first with surface electrode and then by needle electrode; by a Neuropack S1 MEB- 9400^©^ (Nihon Kohden, Japan). 5) For each compound muscle action potential, the latency, amplitude, duration and area of the negative peak of the gastrocnemius and cranial tibial muscles were measured and recorded^5^ ^,^ ^8^ ^,^ ^9^.

BioEstat^©^ 5.3 software was used for analyses. The results were presented as a mean ± standard deviation. The Pearson’s correlation test was used to determine the association between the rat’s weight and temperature *Vs* electroneuromyography parameters. Mann-Whitney U test was used to compare latency, amplitude, duration and area of muscle action potential between the different types of electrode. A significance level of 5% was adopted as cutoff for rejection of the null hypothesis.

## Results

The mean of the animals’ weight was 267.00 ± 1.17g and of the temperature was 34,4 ±0,92ºC. The electroneuromyography analysis of both type electrode of the muscle action potential of the gastrocnemius muscle was described in the table 1. There were no significant differences between the groups in the mean of duration (p=0.21), latency (p=0.34), amplitude (p=0.78) or area of the negative peak (p=0.51). There no correlation between the rats’ weight and temperature *vs.* compound muscle action potential (p>0.05) in gastrocnemius muscle.

The electroneuromyographic analysis of both type electrode of the muscle action potential of the cranial tibial muscle was described in the Table 1. There were no significant differences between the groups in the mean of duration (p=0.70), latency (p=0.49), amplitude (p=0.67) and area of the negative peak (p=0.24). There no correlation between the rat’s weight and temperature *vs.* electroneuromyography parameters (p>0.05)

**Table 1:** Compound muscle action potential analysis of gastrocnemius and cranial tibial muscles.

Analysis	Gastrocnemius muscle		Cranial tibial muscle	
	Needle eletrode	Superfice eletrode	Needle eletrode	Superfice eletrode
Duration	2.32 ±0.19	2.53 ±0.45	2.11 ±0.22	2.23 ±0.53
Latency (ms)	1.21 ±0.17	1.11 ±0.13	1.22 ±0.19	1.09 ±0.18
Amplitude (mV)	32.66 ±22.11	29.14 ±12.31	38.72 ±12.51	31.45 ±22.96
Area (mV.ms)	39.51 ±18.66	32.34 ±25.06	47.23 ±13.64	34.28 ±18.28

## Discussion

 Despite the great standardization of the technique of electroneuromyography application in humans, there are still divergences regarding animals, ranging from the intensity of the stimulus to the position in which the electrodes should be placed, mainly due to anatomical variations that the animals present^4^ ^-^ ^6^. Therefore, there is a need to standardize the various variables for a better comparison between studies, as well as to enable systematic reviews and/or meta-analyzes. Thus, to our knowledge, no studies have evaluated if the type of electrode used affects the electromyoneurography parameters in rats.

Regarding the type of electrode used, the needle electrode presents as an invasive technique, depending on the operator’s experience, it can eventually overpass the muscle to be evaluated and end up presenting results corresponding to another nerve^8^ ^,^ ^10^. However, for the evaluation of large muscle groups, as those mostly assessed, there is little risk of this bias^3^ ^,^ ^4^ ^,^ ^10^. In addition, in this study, a greater amplitude was verified in both muscle groups evaluated; Indicating that through this technique it is possible to better recruit all the muscular group, being this important fact for the realization of studies in nerves related to small muscular groups.

Surface electrodes have the advantage of being a non-invasive method, and can be used with only the sedation of the animals, facilitating the accomplishment of multiple evaluations mainly in follow-up studies^5^ ^,^ ^11^. However, there is a need to know the local anatomy well to avoid overlapping with other muscle groups or to obtain a partial reading of the target muscle group^9^.

It is important to note that the choice of electrodes also depends on the availability of suitable material adapted to the dimensions of the animals as well as previous experience. In addition, there are other types of electrodes such as the hook that requires the nerve to be exposed surgically^11^ ^,^ ^12^.

The gastrocnemius and cranial tibial muscles were chosen because of their known innervation by the tibial and common fibular nerves, respectively branches of the sciatic nerve, which is the most frequently used as the model system of peripheral nerve injury^8^ ^,^ ^9^ ^,^ ^11^ ^,^ ^12^. 

The possibility of comparing the obtained data was feasible due to the standardization of weights and sex of the animals^2^ ^,^ ^5^ ^,^ ^7^ ^,^ ^13^ and the constant calibration and maintenance of temperature above the levels described by Stecker & Baylor^4^, in a study of the effect of temperature on the potential of nervous action, in which only there were alterations of amplitude and area when the animals were submitted to temperatures below 27ºC, and permanent loss of the nervous action potential appeared only after cooling below 10ºC for long periods.

 The findings reinforce that the use of the non-invasive method is safe and reliable, allowing more and more studies with long periods of observation and several points of analysis, fundamental for a better understanding of the process of peripheral nerve recovery^1^ ^,^ ^6^ ^,^ ^14^. On the other hand, the invasive technique maintains its great applicability in studies that require evaluation of the animals in a single time, and, especially, when this is the technique more dominated by the researcher.

The present study has some limitation: It was not evaluated the sensory and mixed nerve conduction^8,15^ and in surgical post-operation (end-to-end^9^ ^,^ ^15^, end-to-side^8^ ^,^ ^11^ ^,^ ^16^ and side-to-side^17^ neurorrhaphy). If the study had been conducted in this scenario, the electrode’s type could affect the results. However, the motor’s nerve is the most frequently used in research^1^ ^,^ ^6^ ^,^ ^14^.

## Conclusion

 The type of electrode (needle *vs.* surface) do not affect the electroneuromyography parameters in gastrocnemius and cranial tibial muscles.
